# Performance of Pit Latrines and Their Herd Protection Against Diarrhea: A Longitudinal Cohort Study in Rural Ethiopia

**DOI:** 10.9745/GHSP-D-22-00541

**Published:** 2024-06-27

**Authors:** Seungman Cha, Sunghoon Jung, Tadesse Abera, Ermias Tadesse Beyene, Wolf-Peter Schmidt, Ian Ross, Yan Jin, Dawit Belew Bizuneh

**Affiliations:** a Department of Disease Control, Faculty of Infectious and Tropical Disease, London School of Hygiene & Tropical Medicine, London, United Kingdom.; b Department of Global Development and Entrepreneurship, Graduate School of Global Development and Entrepreneurship, Handong Global University, Pohang, South Korea.; c Public Health Institute, Addis Ababa, Ethiopia.; d Department of Human Ecology and Technology, Graduate School of Advanced Convergence, Handong Global University, Pohang, South Korea.; e Department of Microbiology, Dongguk University College of Medicine, Gyeongju, Korea.; f Independent Consultant, Addis Ababa, Ethiopia.

## Abstract

In sanitation policies, “improved sanitation” is often broadly described as a goal with little rationale for the minimum standard required. We conducted a secondary analysis of data collected as part of a cluster randomized controlled trial in rural Ethiopia. We compared the performance of well-constructed and poorly constructed pit latrines in reducing child diarrhea. In addition, we explored whether having a well-constructed household latrine provides indirect protection to neighbors if cluster-level coverage reaches a certain threshold. We followed up children aged younger than 5 years (U5C) of 906 households in rural areas of the Gurage zone, Ethiopia, for 10 months after community-led total sanitation interventions. A study-improved latrine was defined as having all the following: pit of ≥2 m depth, slab of any material, drop-hole cover, wall, roof, door, and handwashing facilities (water and soap observed). U5C in households with a study-improved latrine had 54% lower odds of contracting diarrhea than those living in households with a latrine missing 1 or more of the characteristics (adjusted odds ratio [aOR]=0.46; 95% confidence interval [CI]=0.27, 0.81; *P*=.006). Analyses were adjusted for child age and sex, presence of improved water for drinking, and self-reported handwashing at 4 critical times. The odds of having diarrhea among those with an improved latrine based on the World Health Organization/UNICEF Joint Monitoring Program (JMP) definition (i.e., pit latrines with slabs) were not substantially different from those with a JMP-unimproved latrine (aOR=0.99; 95% CI=0.56, 1.79; *P*=.99). Of U5C living in households without a latrine or with a study-unimproved latrine, those in the high-coverage villages were less likely to contract diarrhea than those in low-coverage villages (aOR=0.55; 95% CI=0.35, 0.86; *P*=.008). We recommend that academic studies and routine program monitoring and evaluation should measure more latrine characteristics and evaluate multiple latrine categories instead of making binary comparisons only.

## Abstract (precis)

We believe that the potential of improved sanitation in many existing studies may have been frequently underestimated because the quality was poor and the coverage, particularly of improved latrines, was low or did not reach a sufficient level.

## Teaser

**Key Messages:**

- We compared the performance of a well-constructed latrine with a pit of 2 m or more depth, slab of any material, drop-hole cover, wall, roof, door, and handwashing facilities (water and soap observed) with a poorly constructed one, missing 1 or more of these features or having only a pit latrine with a slab, on interrupting the fecal-oral transmission pathway and reducing child diarrhea.
- Children living in households with a well-constructed latrine were less likely to have diarrhea than those living in households with a poorly constructed one.
- In villages with 50% or more of households having well-constructed latrines and 70% or more of households having a pit latrine with a slab (high coverage), children living in a household with no latrine or a poorly constructed one were less likely to contract diarrhea than those with no latrine or a poorly constructed one in a village with low coverage.

**Key Implications:**

- Policymakers should advocate for universal health coverage of water, sanitation, and hygiene interventions to confer herd protection against disease transmission.
- Future research should investigate the relationship between latrine design and health outcomes.

## INTRODUCTION

Disposing of human excreta into the ground has been practiced for thousands of years.^1^ Proper disposal of excreta improves human health and quality of life, contributing to socioeconomic development.^2^^–^^5^ Pit latrines are the most common form of sanitation in many countries.^6^ In 2017, 3.1 billion people were reported to use improved on-site sanitation facilities, and an estimated 701 million people used unimproved on-site sanitation facilities, including pit latrines without a slab or platform for their excreta disposal.^6^ Pit latrines are a commonly recommended sanitation system for populations likely to be constructing household latrines using locally available and affordable materials.^7^ This is particularly the case in remote rural areas, where community-led total sanitation (CLTS) interventions are being carried out without any material or financial subsidies.^7^

Pit latrines are considered to be the first rung of the sanitation ladder above open defecation, from which people can continue climbing to higher levels of service.^7^ The key reasons for uptake of pit latrines in many low-income countries lie in the following features: pit latrines are simple to construct, do not require flushing water, are easy to operate and maintain, are easy to use for the disposal of various bulky anal cleansing materials, and do not cost a lot.^1^ To dispose of human excreta safely, the pit content should not come into direct contact with humans, insects, or animals.^8^^,^^9^

In accordance with these trends, many sub-Saharan African countries adopted and promoted pit latrines.^10^^,^^11^ A pit latrine with a slab has been considered an improved latrine by UNICEF since 2008. However, some have highlighted the importance of hygienic latrines beyond the “improved sanitation” defined by the Millennium Development Goals.^12^^–^^14^ Against this backdrop, a number of countries in sub-Saharan Africa have adopted policies for sanitation improvements, but there was little emphasis on the minimum standard of pit latrines required for disrupting the transmission of fecal-oral pathogens, with the exception of a few countries.^1^^,^^15^^,^^16^ For example, the Kenya government released a sanitation policy highlighting the importance of accessibility to safe sanitation facilities, which provided a range of sanitation technology options. According to this policy, the minimum requirement is “at least an upgraded pit latrine,” examples of which included “provision of super structures, covering of the pit opening/squat hole with a suitable cover, plastering of the latrine floor with cement and introduction of a vent pipe to improve the hygiene conditions of the latrine.”^17^ In Sudan’s sanitation policy, by contrast, latrine design was not highlighted within specific strategies, although it did outline the sanitation ladder, including improved facilities.^18^

Despite the prevailing view of latrine improvement as an intervention that promotes health, it should be kept in mind that latrines could, in fact, play a role in transmitting disease if they are badly constructed.^19^ For example, some low-quality latrines taken up after CLTS interventions have sometimes been criticized as involving “fixed point open defecation” by collecting excreta in one place nearer the household but still accessible to animals/flies.^20^ In this regard, achieving the open defecation-free status, as it is generally defined, might end up disseminating fixed-point open defecation practices if CLTS implementers are not cautious about latrine design. Thus, we need to understand the minimum standard of pit latrine design for sanitation interventions to help interrupt the transmission of diseases. Although there are different types of pit latrines, it is currently unclear which latrine characteristics help disrupt fecal-oral transmission.^1^^,^^21^^,^^22^ According to a review of the performance of pit latrines, despite their widespread application and use across the globe, the relationship between latrine type or design and performance on health outcomes has not been thoroughly assessed.^1^ Previous studies have mainly focused on latrine coverage, not categorizing latrines by type or design.

Although there are different types of pit latrines, it is currently unclear which latrine characteristics help disrupt fecal-oral transmission.

Meanwhile, sanitation interventions have been thought to provide herd-protective effects.^23^ Herd protection refers to the indirect protection provided to people who did not have a latrine. If herd protection effect exists, children living in a household without a latrine in a village with high latrine coverage are less likely to have diarrhea than those without a latrine in a village with low coverage because having a household latrine provides indirect protection to those who do not have a household latrine in a village with high latrine coverage.

However, this concept has not been thoroughly investigated in the field of sanitation, and empirical studies exploring the herd protection offered by sanitation interventions are scarce.^24^^–^^27^ Some studies have attempted to investigate herd protection against infectious diseases, childhood nutrition, or mortality from drinking water, sanitation, and/or hygiene interventions.^28^^–^^37^ Some studies suggested that sanitation coverage provides an indirect effect against some diseases, such as trachoma and malaria, and on nutritional outcomes. Studies investigating herd protective effects of water and sanitation on child diarrhea are scarce, and few studies examined externalities of sanitation coverage by latrine type.^28–30^^,^^38^^–^^43^

Fuller et al. estimated the herd protection effect of sanitation improvements using hypothetical mathematical modeling.^23^ They highlighted the knowledge gap in empirical research assessing the herd protective effects of sanitation interventions. A recent study on the spill-over effects of sanitation also has pointed out the knowledge gap on the herd protective effects of water and sanitation interventions.^44^ In another recent study by Contreras et al., higher community sanitation coverage was associated with improved child health, including diarrheal reduction, but coverage with exclusively hygienic latrines was not associated with any outcome, which warrants further study.^45^ We aimed to investigate whether relatively well-designed pit latrines conferred greater health benefits than poorly constructed ones. We compared the performance of well-constructed and poorly constructed pit latrines on reducing child diarrhea. We also explored to what extent indicators of fecal-oral transmission pathways, such as the presence of feces or flies around the pit hole, are associated with latrine design or structure. In addition, we explored whether children living in a household without a latrine or with a poorly structured latrine in a village with high coverage are less likely to have diarrhea than those living in a household without a latrine or with a poorly structured latrine in a village with low coverage.

## METHODS

### Study Design and Data Collection

This is a secondary analysis of data collected alongside a cluster randomized controlled trial (cRCT) that was conducted in 2 districts in Ethiopia to investigate the effect of CLTS on child diarrhea. The study protocol of the cRCT was published previously,^46^ as were studies on the health and economic effects of the CLTS intervention.^47^^,^^48^ The trial was conducted from January 2016 to January 2017. The 7-day period prevalence of child diarrhea based on parental reports was assessed 3 months before and 3, 5, 9, and 10 months after the CLTS triggering. The same dataset for evaluating the health and economic effects of the CLTS intervention was used for this study. In total, 906 households enrolled in this study in 2015, representing 25.7% of all households and 80.2% of households with at least 1 child aged younger than 5 years (U5C) in 48 villages. Of those enrolled, 865 (95.5%) were followed up at 12–13 months after enrollment.

### Study Area

The study areas were the Cheha and Enemore Ena Ener Districts, which are located 185 km to the southwest of Addis Ababa, the capital city of Ethiopia. The population of each district was 133,233 and 204,937, respectively, in 2014. Crop production, including coffee, khat, and oil seeds, is the major income source in these districts. Guragenya are the predominant ethnic group, and Muslims and Ethiopian Orthodox Christians comprise 64% and 33% of the population, respectively.

### Sampling and Sample Size

The sample size to design the cRCT was estimated using the formula developed by Hayes and Bennett to design the cRCT study.^49^ The formula produced 48 villages and 1,200 households for the trial. Two-stage sampling was employed to select subjects. Forty-eight villages were selected from 212 villages based on having the lowest water and sanitation coverage before the intervention.^46^ We then listed all the households with at least 1 U5C in 48 villages and selected 25 households from each village using SPSS version 21 (IBM Corp., Armonk, NY, USA) before the baseline survey. We recruited 1,070 households in 48 villages at baseline, which decreased to 906 households before the first round of follow-up because some of the registered children were found to be duplicated or living in the same household.

### Intervention

CLTS activities (pre-triggering, triggering, post-triggering, and open defecation-free declaration and verification) were conducted in 24 intervention villages for 10 months in 2016–2017 (Supplement 1).^46^^,^^47^ Pre-triggering and triggering were conducted in February and March 2016, and open defecation-free declaration and verification were carried out in February 2017. Pre-triggering and triggering took 1 day per village, respectively. Post-triggering activities were done for 10 months after the triggering. CLTS promoters were recruited from every village to mobilize village residents and encourage them to take up household latrines using locally available and affordable materials. No financial or material subsidies were provided to any village residents.

### Analysis

We combined the treatment and control groups and recategorized the households according to the presence and type of a latrine at the household level and coverage per type at the village level, regardless of their allocation results in the trial. In this study, a “study-improved latrine” was defined as having all of the following: a pit of 2 m or more depth, slab of any material, drop-hole cover, wall, roof, door, and handwashing facilities (water and soap observed).^47^^,^^48^ A “study-unimproved latrine” was defined as missing 1 or more of these features. At the same time, we also analyzed the performance of an improved latrine based on the World Health Organization/UNICEF Joint Monitoring Program (JMP) definition, a pit latrine with a slab, which we referred to as a “JMP-improved latrine.” We could not carry out some measurements, including pit depth, fly counts, and feces counts, due to the heavy floods around the second round of the survey (at 5 months) and therefore were unable to categorize latrines as improved or not. Thus, we excluded the second round of data. We assessed the demographic and socioeconomic characteristics of caregivers, household heads, and U5C. Village-wide variables, such as the coverage of improved water access, improved latrines, and handwashing at critical times, were also estimated. Improved latrine and handwashing practices were measured at every round of the household survey. For improved water, the baseline value was analyzed, assuming that it would remain the same for the 10-month follow-up period because there was no intervention for water source improvement during the CLTS intervention period.

Improved water was defined according to the JMP criteria.^6^ For handwashing practices, we defined appropriate handwashing practices as when participants responded, unprompted, that they had washed their hands with soap at all 4 of the following critical times during the previous day: before preparing food, after defecating, before feeding a child, and after cleaning a child’s anus.

#### Primary Outcomes by Latrine Type

First, we compared the diarrhea prevalence of children living in households with a study-improved latrine with those in households with a study-unimproved latrine. We also compared the diarrhea prevalence of children living in households with a study-unimproved latrine with those in households without any latrine. We focused on investigating whether the diarrhea prevalence was different between children according to the presence of a study-improved or study-unimproved latrine in their household. Second, we compared the presence of feces and flies around the pit hole between study-improved and study-unimproved latrines. Feces were counted on the spot by enumerators. Flies were caught by a glue trap of the same length that was put around a pit hole for 30 minutes. Similarly, we assessed latrine utilization using 4 different proxy indicators that were directly observed: the presence of wet feces, a worn path from the house to the latrine, the absence of a spider web at the front part of the latrine, and the presence of odor.

We analyzed village-level coverage of improved water, sanitation, and hygiene practices as categorical variables for the primary analysis, not as continuous variables, because herd protection was expected to occur when the coverage exceeded a certain threshold level, based on previous studies in the literature.^23^ When designing the study protocol, we set the threshold of high coverage at 66%, referring to a previous trial.^37^ In this study, we adjusted the threshold to 50% in terms of the operational definition of improved latrine (study-improved) and 70% in terms of improved latrine according to the JMP definition (JMP-improved) because only a few clusters reached 70% or above at 10 months of follow-up in terms of operational definition of improved latrine (study-improved) in this study. In what follows, “study-improved latrine” refers to the operational definition of an improved latrine in this study, and “JMP-improved latrine” refers to the improved latrine according to JMP criteria. For drinking water and handwashing practices, we also set the threshold at 70%, referring to previous studies.^23^

#### Herd Protection

**FIGURE 1:**
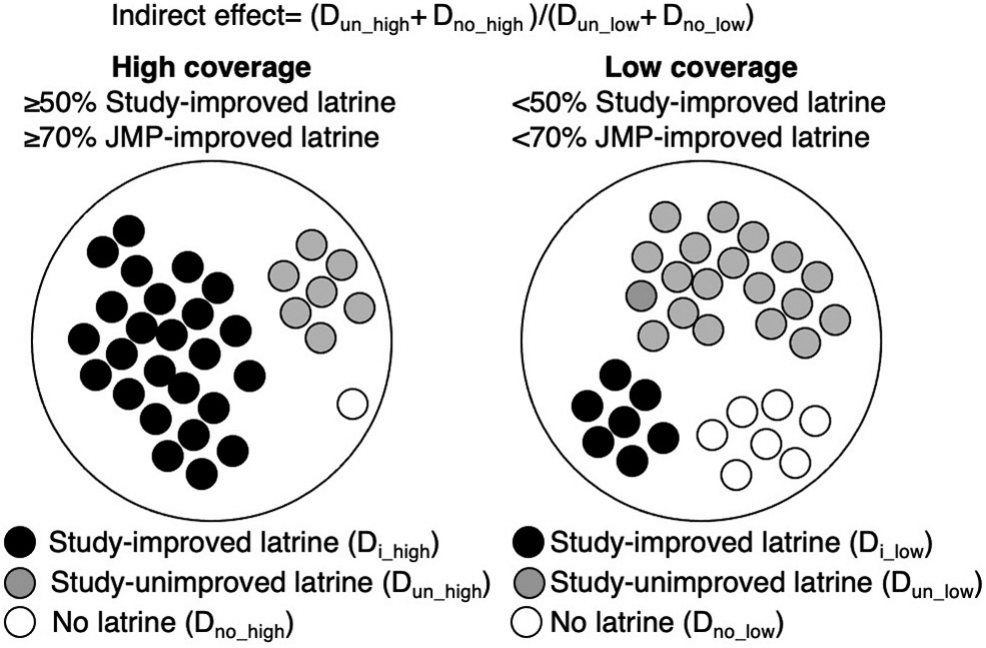
Indirect Effect of Improved Latrines on Risk of Diarrhea in Children Abbreviations: D_i_, risk of diarrhea in children in households that took up improvement sanitation; D_no_, risk of diarrhea in children without any type of latrine; D_un_, risk of diarrhea in children in households that took up a latrine but not an improved one, JMP, World Health Organization/UNICEF Joint Monitoring Program.

To measure herd protection, we followed the framework proposed by Halloran et al.^50^ The direct effect is described as the relative reduction in disease of village members who directly received an intervention compared with those who did not receive the intervention. In their study, the direct effect is denoted by D_i_/D_0_, where D_i_ represents the risk of diarrhea in children in households that took up improved sanitation, and D_0_ represents the risk in those without an improved latrine. Herd protection (indirect effect) is denoted by D_0_high_/D_0_low_, where D_0_high_ represents the risk of diarrhea in the children in households without improved latrine in high-coverage communities, and D_0_low_ represents the risk in those without an improved latrine in low-coverage communities. We separated D_0_ into D_un_ and D_no_, where D_un_ represents the risk of diarrhea in children in households that took up a latrine but not an improved one, and D_no_ represents the risk of diarrhea in those without any type of latrine. We analyzed both D_0_high_/D_0_low_ and D_un_high_/D_un_low_. We could not analyze D_no_high_/D_no_low_ because there were too few households without any latrines in high-coverage communities (Figure 1).

For assessing direct and indirect effects, we used generalized estimating equations to explore a population-averaged effect. For assessing herd protection, we maintained those with an unimproved or no latrine in the dataset while dropping all other subjects and estimated the effect of high coverage in the marginal model. By doing so, we could compare 2 children with an unimproved or no latrine, 1 living in a community of high coverage and the other of low coverage, according to the thresholds previously reported. The same methods were applied also for assessing the direct effect. Exchangeable covariance matrix, log link, and robust standard errors were used for the generalized estimating equations. We adjusted for the key confounding factors, including child age and sex, the presence of improved water for drinking, and appropriate handwashing at 4 critical times (before preparing food, after defecating, before feeding a child, and after cleaning a child’s buttocks).

#### Multilevel Modeling of the Coverage Effect

To further understand the herd protection offered by village-level coverage, multilevel logistic regression analysis was applied, in which repeat observations of the same individual (survey time) were the first level, individuals were the second level, and villages were the third level. We fitted 6 different models. Model 1(level 1 with only time variable) was used as the baseline model to decompose the total variance of diarrhea between the individual and village level. This was selected as the baseline model because an intercept-only model (null model) overestimates the variance at the occasion level and underestimates the variance at the subject level.^51^

Model 2 contained only individual-level factors, whereas model 3 only included village-level variables. We extended these single-level factors to form models 4, 5, and 6 by accommodating individual- and village-level variables. We estimated a fixed slope for the coefficient of an improved latrine in model 5, whereas a random slope was used in model 6.

##### Measures of association (fixed effects)

Odds ratios were measured to assess the associations between individual-level variables and the prevalence of diarrhea with 95% confidence intervals and their *P*-values after adjusting for potential confounders at both the individual and village levels.

##### Measures of variation (random effects)

We explored random effects by assessing village-level variance, the median odds ratio (MOR), intra-cluster correlation, proportional change in variance (as a percentage), and upper and lower interval odds ratios (IORs).^52^^,^^53^

##### Model fitness test

The deviance, defined as −2×LN (likelihood), indicates the model fit of the data, where LN is the natural logarithm and likelihood is the value of the likelihood function at convergence. The lower the deviance, the better the model fits. In this study, all the models we compared were nested, meaning a more general model can derive a more specific model by removing some parameters. In the 2 nested models, the difference in the deviances follows a chi-square distribution. We performed the likelihood ratio test to explore the difference in the deviance between the 2 models.

We used the following equation for estimating the proportional change in community variance:

$$\left({\text{PCV}}_{C}\right):{\text{PCV}}_{C}=\left(V_{C-1}-V_{C-2}\right)/V_{C-1}$$

where V_C-1_ is the community variance in the empty model and V_C-2_ is the community variance in another model. For example, comparing model 1 with model 2, if PCV_C_ is 0.3, then 30% of the community variance in the empty model is attributable to the community factors considered.^52^^,^^53^

### Ethical Approval

We obtained ethical approval from the National Research Ethics Review Committee of the Ethiopian Government (NRERC 3.10/032/2015; July 29, 2015). This trial was registered as an ISRCT (ISRCTN82492848, March 13, 2015). Informed consent for enrollment was obtained from caregivers in written form.

## RESULTS

Table 1 provides both the individual characteristics of household members who participated in this study over 1 year and details on village-wide coverage of improved water, sanitation, and hygiene. At 10 months of follow-up after the CLTS triggering, 166 (19.2%) households in 48 villages had completed the construction of an improved household latrine meeting all the study criteria, and 97 (11.2%) used an improved latrine overall (based on direct observation on wet feces). Overall, the average age of the youngest U5C in the 906 households was 24 months (standard deviation, 16 months). Of 906 household heads, 58% were Muslim and 37% were Christian. Farmers accounted for 80% of household heads’ occupation (data not shown).

**TABLE 1.** Basic Characteristics of Participants and Their Community, Two Districts, Rural Ethiopia

	**Baseline**	**3 months, June 2016**	**9 months, December 2016**	**10 months, January 2017**
**Individual/household variable**				
**Caregiver**				
Age, mean (SD), years	29.7 (5.6)			
Education, % (n/N)				
None	63.8 (578/906)			
1–4 grade completed	12.8 (116/906)			
5–8 grade completed	12.6 (114/906)			
Gender, female	98.5 (892/906)			
**Household head, % (n/N)**				
Ethnicity, Guragenya	95.4 (864/906)			
Religion				
Muslim	58.3 (528/906)			
Christian	37.3 (338/906)			
**Child**				
Age, mean (SD), months	24.2 (15.8)			
Sex, female, % (n/N)	50.3 (456/906)			
Improved water, % (n/N)	73.5 (666/906)			
Improved latrine, % (n/N)	0.3 (3/906)	12.4 (102/822)	15.4 (127/824)	19.2 (166/865)
Handwashing (4 times), % (n/N)	17.8 (162/906)	12.2 (100/822)	19.2 (158/824)	21.4 (185/865)
**Collective variables**				
High coverage of improved water^a^, % (n/N)				
Household	70.6 (640/906)			
Cluster	33.3 (16/48)			
High coverage of study-improved latrine^b^, % (n/N)				
Household^c^	0 (0/906)	6.6 (54/822)	10.6 (87/824)	18.3 (158/865)
Cluster	0.0 (0/48)	8.3 (4/48)	10.4 (5/48)	20.8 (10/48)
50%–59%	0^d^	2	1	4
60%–69%	0	0	1	2
70%–79%	0	1	2	3
80%–89%	0	1	1	1
High coverage of handwashing^e^, % (n/N)				
Household	0.0 (0/906)	6.7 (55/822)	5.6 (46/824)	11.1 (96/865)
Cluster	0.0 (0/48)	8.3 (4/48)	6.3 (3/48)	12.5 (6/48)

Abbreviation: SD, standard deviation.

^a^ High coverage is 70% or more of improved water (piped water into dwelling, plot or yard; public tap/standpipe; tube well/borehole; protected dug well; protected spring; and rainwater).

^b^ High coverage is 50% or more of improved latrine, defined as having a pit of ≥2m depth, slab of any material, drop-hole cover, wall, roof, door, and handwashing facilities (water and soap observed).

^c^ Households in the villages of high coverage of a study-improved latrine.

^d^ Number of villages in each category of coverage.

^e^ High coverage is 70% or more handwashing (washing hands at before preparing food, after defecating, before feeding a child, and after cleaning a child’s buttocks).

The majority of caregivers had not graduated from primary school. At baseline, only a small proportion of people (17.8%) reported they washed their hands with soap at all 4 critical times (after defecating, before food preparation, after cleaning child’s buttocks, and before feeding child). There were 10 of 48 villages with an improved latrine coverage of 50% or above at 10 months after the triggering. The number of households in the high-coverage group (i.e., 50% or above in terms of study-improved latrine) was 0 at baseline but reached 158 (18.3%) at 10 months. The number of villages with a high coverage level of improved water was 16 of 48 villages at baseline, and we assumed that this figure would remain unchanged because no interventions were done during the trial.

**FIGURE 2:**
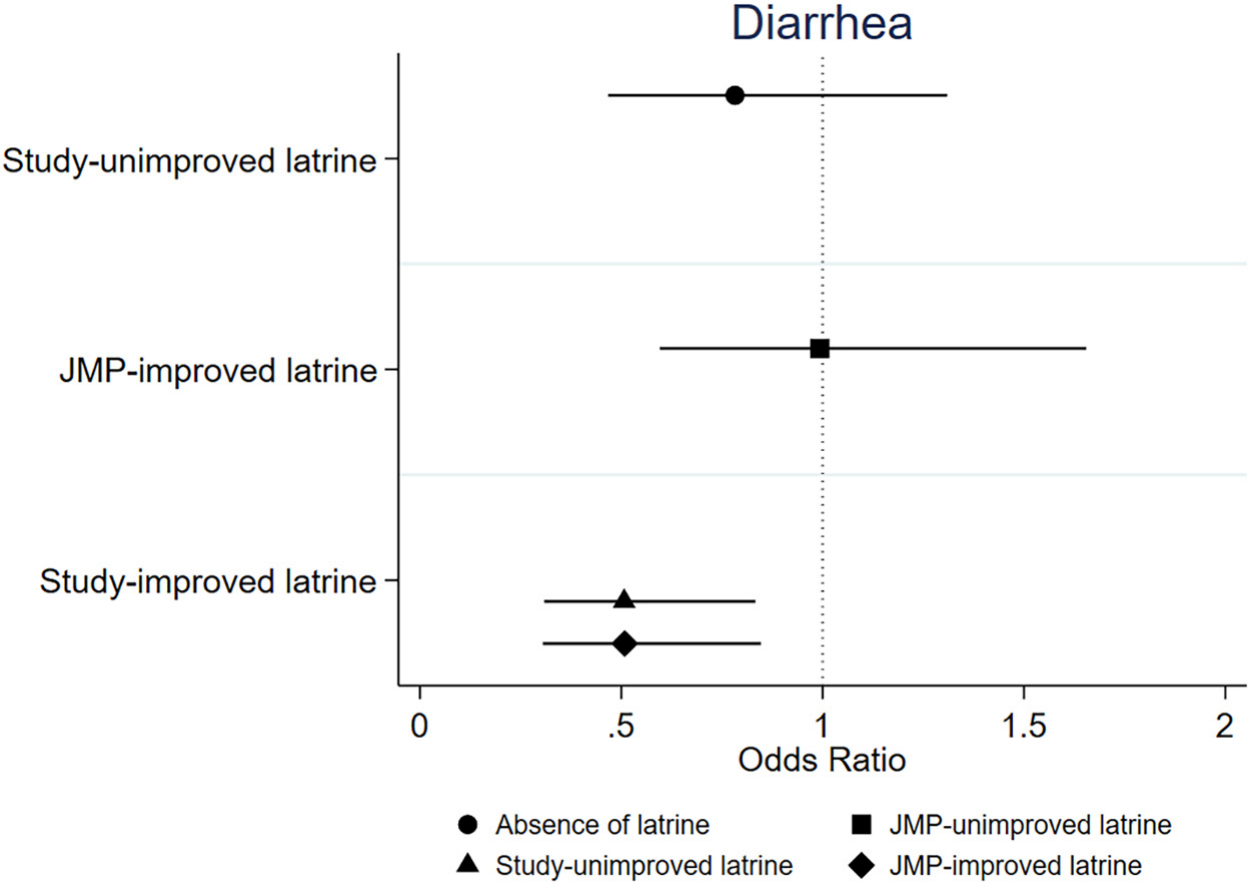
Performance of a Latrine on Child Diarrheal Prevalence by Type of Latrine Abbreviation: JMP, World Health Organization/UNICEF Joint Monitoring Program.

Table 2 shows that children living in households with access to a study-improved latrine were less likely to contract diarrhea than their counterparts with a study-unimproved latrine. The U5C in households with a study-improved latrine had an over 50% lower odds of contracting diarrhea than those living in households with a study-unimproved latrine adjusting for child age and sex, the presence of improved water for drinking, and appropriate handwashing at 4 critical times (adjusted odds ratio [aOR]=0.46; 95% confidence interval [CI]=0.27, 0.81; *P*=.006) The aOR of contracting diarrhea among children living in households with a study-unimproved latrine compared with those without any latrine indicated a smaller reduction in the odds (aOR=0.76; 95% CI=0.40, 1.44; *P*=.40). Supplement Table S1 shows that the odds of having diarrhea among those with a JMP-improved latrine were not significantly different from those with a JMP-unimproved latrines that did not meet the criteria of improved latrine based on JMP definition (aOR=0.99; 95% CI=0.56, 1.79; *P*=.99) (Figure 2).

**TABLE 2.** Performance of Latrines on Child Diarrheal Prevalence by Type of Latrine

		**Absence of Latrine** ^a^	**Presence of Latrine, but Not a Study-Unimproved One** ^b^		**Presence of a Study-Improved Latrine**	
			**Unadjusted**	**Adjusted**	**Unadjusted**	**Adjusted**
All^c^	aOR (95% CI)	1.0	0.73 (0.39, 1.39)	0.76 (0.40, 1.44)	0.46 (0.26, 0.80)	0.46 (0.27, 0.81)
	*P*-value^d^	—	.34	.40	.006	.006
3 months, June 2016	% (n/N)	17.54 (10/57)	15.08 (100/663)	15.08 (100/663)	7.84 (8/102)	7.84 (8/102)
	aOR (95% CI)	—	0.91 (0.19, 4.29)	1.33 (0.26, 6.79)	0.27 (0.05, 1.32)	0.26 (0.04, 1.51)
	*P*-value	—	.91	.73	.11	.13
9 months, December 2016	% (n/N)	21.43 (9/42)	11.60 (76/655)	11.60 (76/655)	6.30 (8/127)	6.30 (8/127)
	aOR (95% CI)	—	0.23 (0.02, 2.36)	0.20 (0.02, 2.18)	0.50 (0.03, 8.83)	
	*P*-value	—	0.22	0.19	0.64	
10 months, January 2017	% (n/N)	17.07 (7/41)	9.42 (62/658)	9.42 (62/658)	4.22 (7/166)	4.22 (7/166)
	aOR (95% CI)	—	0.66 (0.22, 1.95)	0.68 (0.24, 1.95)		
	*P*-value	—	.45	.48		

Abbreviations: CI, confidence interval; aOR, adjusted odds ratio.

^a^ Reference: absence of latrine (adjusted for individual variables: child’s age and sex, presence of an improved water source, handwashing behavior at 4 critical times).

^b^ Reference: presence of a latrine but not a study-improved one (adjusted for individual variables: child’s age and sex, presence of improved water source, handwashing behavior at 4 critical times).

^c^ All the data of June, December, and January were pooled.

^d^ Blanks in the crude and adjusted analysis: the regression model did not converge.

As shown in Table 3, study-improved latrines also showed better performance for the transmission pathway of fecal-oral contamination. The odds of the presence of flies around the pit hole were much smaller in study-improved latrines than in study-unimproved latrines, and the same was true for the presence of feces around the pit hole. Compared to poorly constructed latrines, better latrines showed lower odds of the presence of feces and flies around the pit hole (aOR=0.50; 95% CI=0.33, 0.75; *P*=.001; aOR=0.05; 95% CI=0.03, 0.10; *P*<.001, respectively). For latrine use, we detected no significant difference between those who had a study-improved latrine and those who did not regarding the 4 proxy indicators (Table 4).

**TABLE 3.** Performance of Latrines on Transmission Pathways by Type of Latrine

		**Presence of Feces Around Pit-Hole**		**Presence of Flies Around Pit-Hole**		**Number of Flies**	
		**Presence of Study-Unimproved Latrine**	**A Study-Improved Latrine**	**Presence of Study-Unimproved Latrine**	**A Study-Improved Latrine**	**Presence of Study-Unimproved Latrine**	**A Study-Improved Latrine**
All	aOR (95% CI)	1.0 (reference)	0.50 (0.33, 0.75)	1.0 (reference)	0.05 (0.03, 0.10)	1.0 (references)	–0.35 (–0.40, –0.29)
	*P*-value		.001		<.001		<.001
3 months, June 2016^a^	% (n/N)	241/660	27/102				
	%	36.5%	26.5%				
	aOR (95% CI)		0.60 (0.04−8.67)				
	*P*-value		.71				
9 months, December 2016	% (n/N)	23.4 (153/655)	19.0 (19/127)	83.5 (545/653)	48.4 (61/126)	10.6 (0.6)	2.1 (0.4)
	Mean (SD)					10.6 (0.6)	2.1 (0.4)
	aOR (95% CI)		0.45 (0.16, 1.24)		0.05 (0.01, 0.30)		–0.34 (–0.42, –0.27)
	*P*-value		.12		.001		<.001
10 months, January 2017	% (n/N)	22.6 (149/658)	8.4 (14/166)	83.0 (546/658)	32.5 (54/166)		
	Mean (SD)					6.9 (0.3)	1.5 (0.3)
	aOR (95% CI)		0.25 (0.06, 0.94)		0.16 (0.11, 0.23)		–0.41 (–0.49, –0.34)
	*P*-value		.04		<.001		<.001

Abbreviations: CI, confidence interval; aOR, adjusted odds ratio; SD, standard deviation.

^a^ Blanks in some column in June: flies were not counted.

**TABLE 4.** Latrine Use by Type of Latrine

		**Wet Feces**		**No Spider Web**		**Worn Path**		**Odor** ^a^	
		**Presence of Study-Unimproved Latrine**	**Presence of Study-Improved Latrine**	**Presence of Study-Unimproved Latrine**	**Presence of Study-Improved Latrine**	**Presence of Study-Unimproved Latrine**	**Presence of Study-Improved Latrine**	**Presence of Study-Unimproved Latrine**	**Presence of Study-Improved Latrine**
3 months, June 2016	% (n/N)	45.3 (300/663)	57.8 (59/102)	45.3 (300/663)	59/102	45.3 (300/663)	57.8 (59/102)		
	OR (95% CI)		1.07 (0.53, 2.15)		1.06 (0.53, 2.15)		1.07 (0.53, 2.16)		
	*P*-value		.85		.85		.85		
9 months, December 2016	% (n/N)	64.3 (421/655)	68.5 (87/127)	65.5 (429/655)	63.0 (80/127)	84.7 (555/655)	83.5 (106/127)	70.4 (461/655)	65.4 (83/127)
	OR (95% CI)		1.22 (0.38, 3.96)		0.56 (0.21, 1.54)		0.84 (0.33, 2.13)		0.76 (0.15, 3.76)
	*P*-value		.74		.27		.71		.74
10 months, January 2017	% (n/N)	63.2 (416/658)	58.4 (97/166)	78.7 (518/658)	59.6 (98/166)	92.9 (611/658)	68.1 (113/166)	85.3 (561/658)	62.7 (104/166)
	OR (95% CI)		1.52 (0.61, 3.78)		0.47 (0.16, 1.42)		0.55 (0.11, 2.77)		0.39 (0.14, 1.07)
	*P*-value		.36		.18		.47		.07

Abbreviations: CI, confidence interval; OR, odds ratio.

^a^ Odor was not measured in June.

We divided the 48 villages into high-coverage and low-coverage groups. Table 5 shows the magnitude of the indirect effect (herd protection) and direct effect of a study-improved latrine. Of the children living in households without a latrine or with a study-unimproved latrine, those in the high-coverage villages (70% or more coverage of a JMP-improved latrine) were less likely to contract diarrhea than those in low-coverage villages (aOR=0.55; 95% CI=0.35, 0.86; *P*=.008). The odds of contracting diarrhea among children who lived in a household with a study-improved latrine in high-coverage areas were 67% lower than those of children who lived in a household with a study-unimproved latrine in a low-coverage area (aOR=0.33; 95% CI=0.14, 0.79; *P*=.01).

**TABLE 5.** Magnitude of Indirect Effect and Direct Effect of Study-Improved Latrine

		**Low coverage**		**High coverage**			**Comparison of study-unimproved/no latrine in high- and low-coverage areas**		**Comparison of study-improved latrine in high-coverage areas and all others** ^a^	
		**Absence of a latrine**	**Presence of a latrine, but not an improved one**	**Absence of a latrine**	**Presence of a latrine; but not an improved one**	**Improved latrine**	**aOR (95% CI)**	***P*-Value**	**aOR (95% CI)**	***P*-Value**
All							0.55 (0.35, 0.86)	.008	0.33 (0.14, 0.79)	.01
June (3 months)	% (n/N)	20.5 (8/39)	17.6 (55/312)	11.1 (2/18)	12.8 (45/351)	9.0 (7/78)	0.68 (0.44, 1.04)	.08	0.58 (0.28, 1.19)	.14
December (9 months)	% (n/N)	36.4 (4/11)	15.0 (3/20)	16.1 (5/31)	11.5 (73/635)	6.3 (8/127)	0.68 (0.29, 1.60)	.37	0.27 (0.09, 0.78)	.02
January (10 months)	% (n/N)	50.0 (3/6)	23.1 (3/13)	11.4 (4/35)	9.1 (59/645)	4.2 (7/166)	0.40 (0.26, 0.62)	<.001	0.22 (0.06, 0.85)	.03

Abbreviations: CI, confidence interval; aOR, adjusted odds ratio.

^a^ Study-unimproved or no latrine in low-coverage areas, based on 70% coverage of Joint Monitoring Program improved latrine.

We found similar pattern for direct and indirect effects when we changed the definition of high-coverage areas to “communities with the coverage of 50% or above in terms of a study-improved latrine” although we found no statistical difference (Supplement Table S2).

Table 6 shows the analysis results of multilevel models. Based on the model fit test, the model containing both individual- and village-level variables had the best fit, and model 6 with a random slope for an improved latrine was finally selected. Based on the results of fixed effects, when comparing 2 children with similar predicted risk, 1 living in a community of higher latrine coverage and the other of lower coverage, the odds of having diarrhea decreased by 62% for the former (95% CI=6%, 84%). However, this model could explain only 7% of the variance in diarrhea in the baseline model at the cluster level (proportional change in variance of model 6 compared with model 1), and the IOR-80% for diarrhea was large, from 0.34 to 6.22. According to the results in the random effects, when comparing the odds of 2 randomly chosen children having diarrhea (1 from a high-coverage community and the other from a low-coverage community), the middle 80% of the odds ratio will lie between 0.34 and 6.22. The MOR quantifying the variation between communities by comparing 2 persons from 2 randomly chosen, different communities was 2.14, suggesting there are considerable between-community variations.

**TABLE 6.** Results of Multilevel Analysis

	**Model 1**	**Model 2**	**Model 3**	**Model 4**	**Model 5**	**Model 6**
	**Empty Model**	**Individual-Level Variables**	**Community-Level Variables**	**Individual and Community-Level Variables**	**Individual and Community-Level Variables**	**Individual and Community-Level Variables**
Fixed parts				fixed slope	fixed slope	random slope
Predictor, OR (95% CI)						
Intercept	0.13 (0.08, 0.22)	0.48 (0.18, 1.28)	0.19 (0.09, 0.41)	0.47 (0.18, 1.25)	0.60 (0.20, 1.77)	0.36 (0.17, 1.23)
Time	0.81 (0.73, 0.91)	0.81 (0.73, 0.91)	0.84 (0.75, 0.94)	0.67 (0.55, 0.81)	0.67 (0.55, 0.82)	0.67 (0.5, 0.81)
Study, improved latrine		0.48 (0.27, 0.83)		0.60 (0.33, 1.07)	0.60 (0.33, 1.08)	0.40 (0.15, 1.13)
Improved water		0.88 (0.55, 1.41)		0.87 (0.54, 1.40)	0.95 (0.58, 1.56)	0.85 (0.52, 1.38)
Handwashing		1.03 (0.63, 1.69)		1.05 (0.64, 1.72)	1.11 (0.64, 1.91)	1.06 (0.63, 1.75)
Child’s sex		1.41 (0.89, 2.25)		1.44 (0.90, 2.29)	1.45 (0.91, 2.31)	1.47 (0.91, 2.36)
Child’s age		0.97 (0.96, 0.99)		0.97 (0.96, 0.99)	0.97 (0.96, 0.99)	0.97 (0.96, 0.99)
Coverage of study-improved latrines			0.43 (0.19, 0.98)	0.43 (0.19, 0.97)	0.43 (0.19, 0.98)	0.38 (0.16, 0.94)
Coverage of improved water			-	0.67 (0.31, 1.42)	-	-
Coverage of handwashing			-	0.90 (0.46, 1.75)	-	-
Random parts						
Cluster-level variance	0.86 (0.15)	0.82 (0.14)	0.79 (0.14)	0.79 (0.14)	0.80 (0.14)	0.80 (0.14)
Individual-level variance	1.16 (0.16)	1.06 (0.16)	1.18 (0.16)	1.07 (0.16)	1.07 (0.16)	1.12 (0.17)
ICC-VPC	0.18 (ICC)	0.17 (ICC)	0.16 (VPC)	0.43 (VPC)	0.43 (VPC)	0.42 (VPC)
Explained variation	Ref (cluster)	4.7%	8.1%	9.3%	7.0%	7.0%
(i.e., PCV in %) proportional change in variance by the new model	Ref (individual)	8.6%	-	8.6%	7.8%	3.8%
Deviance^a^	1667.4	1644.6	1657.5	1639.1	1640.3	1635.1
Model fit test results,^b^ chi-square (*P*-value)	-	22.81 (*P*<.001)	-	5.52 (*P*=.14)	4.28 (*P*=.04)	5.22 (*P*=.02)
MOR	2.26	2.18	2.12	2.12	2.17	2.14
IOR upper, lower			6.42, 0.37	6.42, 0.37	6.54, 0.36	6.22, 0.34

Abbreviations: ICC, intra-cluster correlation; IOR, interval odds ratio; MOR, median odds ratio; PCV, proportional change in variance; VPC, variance partition coefficient.

^a^ The deviance: –2 × LN (likelihood), where likelihood is the value of the likelihood function at convergence, and LN is the natural logarithm.

^b^ The likelihood ratio test (Model 4 of lower deviance was compared with Model 2 of larger value, which was not significantly different (*P*=.14). Model 5 of lower deviance was compared with Model 2 of larger deviance, which was significantly different (*P*=.04). Model 6 has the lowest value of deviance was compared with Model 5, which was significantly different (*P*=.02). Hence, we finally selected Model 6.

## DISCUSSION

This study suggests that children living in households with a study-improved latrine were less likely to have diarrhea than those with a study-unimproved latrine and those with a JMP-improved latrine. In addition, study-improved latrines had herd-protective effects when the level of coverage was high (study-improved latrine coverage was 50% or more). Children living in a household without a latrine or with a study-unimproved latrine in a village with high coverage were less likely to contract diarrhea than those without a latrine or with a study-unimproved latrine in a village with low coverage. The 2 attributes most commonly missing from JMP-improved latrines that prevented them from being categorized as “study-improved” were drop-hole cover and pit-depth at 10 months (Supplement Table S3).

Study-improved latrines had herd-protective effects when the level of coverage was high.

Latrine use was not substantially different between members of households with a study-improved and study-unimproved latrine based on direct observations. In this regard, a possible explanation for the lower odds of contracting diarrhea among children living in a household with a study-improved latrine than in those living in a household with a study-unimproved latrine could be a reduction in the chances of direct contact with feces via hands or feet or indirect contact via flies inside or around the latrine due to the improved status of a latrine rather than increased latrine use. We found that the odds of feces or fly presence around the pit hole were consistently lower in study-improved latrines than in study-unimproved latrines. Similarly, the number of flies was also lower in study-improved latrines than in study-unimproved ones.

The importance of an improved latrine, even relative to other types of pit latrines, has been highlighted in several studies.^1^^,^^22^ The finding that study-improved latrines had more health benefits than study-unimproved latrines in the category of pit latrines is consistent with a previous study done in the Democratic Republic of Congo.^54^ Nakagiri et al. investigated the association between diarrhea and each specific component of a latrine, such as the pit depth, slab, pit-hole cover, and wall.^22^ According to their study, pit depth and the presence of a slab were associated with diarrhea reduction by directly disrupting fecal-oral transmission. The herd-protective effect of the sanitation intervention was consistent with previous simulation modeling studies.^23^

We could not overcome the typical limitations of the 7-day period prevalence of diarrhea ascertainment solely based on caregivers’ reports, which entails several biases, such as reporting bias, recall bias, and social desirability bias.

For latrine use, we used observation results using 4 different proxy indicators; however, we cannot rule out any possibility that the 4 different proxy indicators may not fully represent their actual use of a latrine. Further research is needed to determine to what extent these indicators adequately represent the actual use of latrines.

Measuring latrine use continues to be a challenge. Efforts to use electronic motion sensors have shown promise in a study in Orissa, but implementation is costly.^55^ A low-cost measurement method of assessing latrine use needs to be developed to be employed at a large scale and a lower cost, for example by using survey tools that camouflage the true purpose of a study measuring latrine use.^56^

The fact that we could not detect significant differences in 4 distinct indicators of latrine use indicates that the possible explanation for the better health benefits of a study-improved latrine lies in the improved status of the latrine structure rather than in increased household latrine use alone.

In this study, we used IOR and MOR because the usual odds ratio interpretation is incorrect for quantifying associations between variables at the cluster level and outcomes at the individual level. The variable of interest, community-level coverage, does not vary between individuals within the community, and we thus have to compare persons with different random effects. The IOR indicates the interval that 80% of odds ratios of having diarrhea lie between 2 randomly chosen children with identical individual covariates, 1 from a high-coverage village and the other from a low-coverage village. The interval contains 1, suggesting that the effect of coverage is small compared to the cluster variability. The MOR quantifies the variation between villages by comparing 2 children with the same covariates from 2 randomly chosen different villages. The MOR in this study was 2.14, which suggests that if a child moves to another village with a higher probability of having diarrhea, the risk of contracting diarrhea will increase 2.14 times. The final model in the multilevel regression analysis explained only a small percentage of the variance in diarrhea at the village level. This points to the fact that there is still large unexplained variance in child diarrhea at the village level in our final model. We could not measure water quality, and handwashing behavior was based on self-report. We infer that the unexplained variance at the village level might have been reduced if we could have included properly measured coverage of water quality and handwashing behavior. If the coverage of a study-improved latrine reached universal coverage, we could explain more variance of child diarrhea at the village level, which warrants further study.

We excluded coverage of improved water and handwashing in the final model based on the model fit test results. Caution is needed when interpreting the final model with no context variables of water and handwashing. This study may not suggest that water and handwashing coverage does not matter. The reason that the final model does not include water and handwashing coverage may probably lie in the method of measurement method of improved water and handwashing. We could not measure the coverage of clean water based on water quality at the point of use. In addition, we relied on respondent’s self-report for handwashing behavior instead of direct observation. If we had measured coverage of clean water based on water quality at the point of use and observed handwashing behavior instead of relying on interviewees’ recall to estimate coverage of handwashing, the final model might have included community-level water and handwashing behavior coverage, which also still need further research.

We assumed that improved water coverage remained the same, as the observation period after CLTS triggering in the trial was only 10 months, and there was no water project in the study area. However, we could not rule out any possibility that it could get better or worse, which was not reflected in our study.

For confounding variables in the adjusted analysis, we referred to previous studies^57^^,^^58^ that modeled risk categories to predict child diarrhea that suggested that socioeconomic characteristics affect diarrhea indirectly via water, sanitation/environment, and hygiene/food. We controlled for child age, sex, water, and handwashing behaviors in the adjusted analysis, but we could not include food hygiene and childcare-related variables due to the absence of data, which is a limitation of this study.

We believe that this study has policy implications in terms of advocating for achieving universal health coverage of water, sanitation, and hygiene. This study also suggests that the potential of “improved sanitation” in many existing studies may have been frequently underestimated because the quality was poor and the coverage, particularly of improved latrines, was low or did not reach a sufficient level in many trials.^59^^–^^62^

This study suggests that the potential of “improved sanitation” in many studies may have been frequently underestimated because the quality of improved latrines was poor and the coverage was low or did not reach a sufficient level in many trials.

We recommend that academic studies and routine monitoring and evaluation programs should measure more latrine characteristics and compare multiple latrine categories instead of just binary comparisons.

The definition of an “improved latrine” should be revisited, at least in the research domain, with a focus on gathering more substantial evidence through rigorous investigation. This is to ascertain whether sanitation facilities can effectively contain feces to prevent fecal contamination. The revised definition should emphasize the latrine’s performance or functionality in interrupting transmission. Consequently, some latrines currently classified as “improved latrines” might need to be reclassified as “unimproved.”

In numerous sanitation interventions, particularly those involving CLTS, the importance of latrine quality appears to have been neglected. Until now, the emphasis on latrine quality or design has not been adequately addressed. In fact, a key principle in the conventional CLTS approach is not to make suggestions regarding the latrine design. Dr. Kamar Kar, the founder of CLTS, argued that placing emphasis on latrine design could lead to issues of inequality. He suggested that the most vulnerable individuals within a community could become further marginalized due to their difficulty in accessing higher-quality latrines.^7^ His concern is understandable, as superior latrine facilities might incur costs that these vulnerable individuals cannot afford. However, if community members cannot reap the benefits of a latrine, it is uncertain whether they would be motivated to continue their climb up the sanitation ladder. Instead, one could deduce that if they experience no advantages from using a latrine, they might revert to their previous practices of open defecation.^63^

Patil et al. argued that sanitation remains beneficial, even if it does not have a direct effect on health, due to its other social benefits, which might imply that the quality of the latrine is of lesser importance.^60^ Ross et al. reported that a sanitation intervention increased the quality of life in low-income settings.^5^ This claim warrants further empirical research in different settings to confirm whether the proposed social benefits extend to low-quality latrines deemed sufficient in many CLTS interventions.

We need to find better ways to roll out sanitation interventions that can deliver high-quality toilets, which interventions focusing on behavior change seem unable to do.

Humphrey et al. advocated new and innovative interventions “that are less dependent on behavior change and more efficacious in reducing fecal exposure—a paradigm shift away from how rural WASH programs are delivered.”^64^ Given the low compliance rate with current sanitation interventions that emphasize behavior change, we may need to rethink these interventions and seek an appropriate approach toward achieving universal sanitation coverage.

## CONCLUSION

This empirical study demonstrated the existence of herd protection from a sanitation intervention and confirmed the importance of reaching universal coverage for water, sanitation, and hygiene. We recommend that academic studies and routine program monitoring and evaluation measure more latrine characteristics and evaluate multiple latrine categories instead of making binary comparisons only. Future research should investigate the relationship between latrine design and health outcomes.

## Supplementary Material

GHSP-D-22-00541_supplement.pdf

## Notes

**Peer Reviewed**

**First published online:** June 4, 2024.

## References

[1] Nakagiri A, Niwagaba CB, Nyenje PM, Kulabako RN, Tumuhairwe JB, Kansiime F. Are pit latrines in urban areas of Sub-Saharan Africa performing? A review of usage, filling, insects and odour nuisances. BMC Public Health. 2016;16:120. 10.1186/s12889-016-2772-z. 26846125 PMC4743102

[2] Freachem RG, Bradley DJ, Garelick H, Mara DD. Sanitation and Disease: Health Aspects of Excreta and Wastewater Management. World Bank; 2010. Accessed May 10, 2024. https://documents1.worldbank.org/curated/en/704041468740420118/pdf/multi0page.pdf

[3] Van Minh H and Nguyen-Viet H, Economic aspects of sanitation in developing countries. Environ Health Insights. 2011;5:63–70. 10.4137/ehi.s8199. 22084575 PMC3212862

[4] Wolf J, Hubbard S, Brauer M, et al. Effectiveness of interventions to improve drinking water, sanitation, and handwashing with soap on risk of diarrhoeal disease in children in low-income and middle-income settings: a systematic review and meta-analysis. Lancet. 2022 Jul 2;400(10345):48–59. 10.1016/s0140-6736(22)00937-0. 35780792 PMC9251635

[5] Ross I, Greco G, Adriano Z, et al. Impact of a sanitation intervention on quality of life and mental well-being in low-income urban neighbourhoods of Maputo, Mozambique: an observational study. BMJ Open. 2022;12(10):e062517. 10.1136/bmjopen-2022-062517. 36195460 PMC9558791

[6] World Health Organization/United Nations Children’s Fund (WHO/UNICEF). Progress on Household Drinking Water, Sanitation and Hygiene 2000-2020, Five Years Into the SDGs. WHO/UNICEF; 2021. Accessed May 10, 2024. https://www.who.int/publications/i/item/9789240030848

[7] Kar K. *Scaling-up Community-Led Total Sanitation: From Village to Nation.* Practical Action Publishing; 2019.

[8] Wagner EG, Lanoix JN. Excreta disposal for rural areas and small communities. Monogr Ser World Health Organ. 1958;39:1–182. 13581743

[9] WHO Study Group on Technology for Water Supply and Sanitation in Developing Countries; World Health Organization. Technology for Water Supply and Sanitation in Developing Countries: Report of a WHO Study Group [Meeting Held in Geneva From 14 to 19 April 1986]. World Health Organization; 1987. Accessed May 10, 2024. https://iris.who.int/handle/10665/38110 3107220

[10] Kalbermatten JM, Julius DS, Gunnerson CG, Mara DD. Appropriate Sanitation Alternatives: a Planning and Design Manual. Johns Hopkins University Press; 1982. Accessed May 10, 2024. https://documents1.worldbank.org/curated/ar/701511468740361506/pdf/multi-page.pdf

[11] Black M. *Learning What Works: A 20 Year Retrespective View on International Water and Sanitation Cooperation: 1978-1998.* World Bank; 1998.

[12] Jenkins MW, Cumming O, Scott B, Cairncross S. Beyond ‘improved’ towards ‘safe and sustainable’ urban sanitation: assessing the design, management and functionality of sanitation in poor communities of Dar es Salaam, Tanzania. J Water Sanit Hyg Dev. 2014;4(1):131–141. 10.2166/washdev.2013.180

[13] Günther I, Niwagaba BC, Lüthi C, Horst A, Mosler H-J, Tumwebaze KI. When Is Shared Sanitation Improved Sanitation? The Correlation Between Number of Users and Toilet Hygiene. Swiss Federal Institute of Technology Zurich; 2012. Accessed May 10, 2024. https://www.dora.lib4ri.ch/eawag/islandora/object/eawag%3A8913/datastream/PDF/G%C3%BCnther-2012-When_is_shared_sanitation_improved-%28published_version%29.pdf

[14] Kwiringira J, Atekyereza P, Niwagaba C, Günther I. Descending the sanitation ladder in urban Uganda: evidence from Kampala Slums. BMC Public Health. 2014;14:624. 10.1186/1471-2458-14-624. 24948084 PMC4071028

[15] Potter A, Klutse A, Snehalatha M, et al. Assessing Sanitation Service Levels. IRC International Water and Sanitation Centre; 2011. Accessed May 10, 2024. https://www.susana.org/en/knowledge-hub/resources-and-publications/library/details/1332#

[16] Water Engineering and Development Center (WEDC). Comparing national sanitation policy content. An initial review of nine country profiles. WEDC; 2005. Accessed May 10, 2024. https://assets.publishing.service.gov.uk/media/57a08c6ae5274a27b20011b5/R8163-Polreview.pdf

[17] Republic of Kenya. Ministry of Health (MOH). *Environmental Sanitation and Hygiene Policy 2016–2030*. MOH; 2016. Accessed May 10, 2024. https://faolex.fao.org/docs/pdf/ken179039.pdf

[18] Republic of Sudan. Federal Ministry of Health (FMOH). National Sanitation and Hygiene Strategic Framework. FMOH; 2016. Accessed May 10, 2024. https://www.unicef.org/sudan/media/1026/file/National-Sanitation-Hygiene-Strategic-Framework-2016.pdf

[19] Myers J. The long-term safe management of rural shit. In: Bongartz P., Vernon N. and Fox J., eds. Sustainable Sanitation for All: Experiences, Challenges, and Innovations. Practical Action Publishing; 2016. Accessed May 10, 2024. https://sanitationlearninghub.org/resource/the-long-term-safe-management-of-rural-shit/

[20] Kar K, Chambers R. Handbook on community-led total sanitation. Plan UK; 2008.

[21] Water Utility Partnership for Capacity Building Africa (WUP Africa). Better Water and Sanitation for the Urban Poor: Good Practice from Sub-Saharan Africa. European Communities and Water Utility Partnership; 2003. Accessed May 10, 2024. https://documents.worldbank.org/en/publication/documents-reports/documentdetail/598951467990330016/better-water-and-sanitation-for-the-urban-poor-good-practice-from-sub-saharan-africa

[22] Nakagiri A, Kulabako RN, Nyenje PM, Tumuhairwe JB, Niwagaba CB, Kansiime F. Performance of pit latrines in urban poor areas: a case of Kampala, Uganda. Habitat Int. 2015;49:529–537. 10.1016/j.habitatint.2015.07.005

[23] Fuller JA, Eisenberg JN. Herd protection from drinking water, sanitation, and hygiene interventions. Am J Trop Med Hyg. 2016 Nov 2;95(5):1201–1210. 10.4269/ajtmh.15-0677. 27601516 PMC5094239

[24] Fewtrell L, Kaufmann RB, Kay D, Enanoria W, Haller L, Colford JM Jr. Water, sanitation, and hygiene interventions to reduce diarrhoea in less developed countries: a systematic review and meta-analysis. Lancet Infect Dis. 2005;5(1):42–52. 10.1016/s1473-3099(04)01253-8. 15620560

[25] Freeman MC, Garn JV, Sclar GD, et al. The impact of sanitation on infectious disease and nutritional status: a systematic review and meta-analysis. Int J Hyg Environ Health. 2017;220(6):928–949. 10.1016/j.ijheh.2017.05.007. 28602619

[26] Venkataramanan V, Crocker J, Karon A, Bartram J. Community-led total sanitation: a mixed-methods systematic review of evidence and its quality. Environ Health Perspect. 2018;126(2):026001. 10.1289/ehp1965. 29398655 PMC6066338

[27] Wolf J, Prüss-Ustün A, Cumming O. Assessing the impact of drinking water and sanitation on diarrhoeal disease in low- and middle-income settings: systematic review and meta-regression. Trop Med Int Health. 2014;19(8):928–942. 10.1111/tmi.12331. 24811732

[28] Barreto ML, Genser B, Strina A. Effect of city-wide sanitation programme on reduction in rate of childhood diarrhoea in northeast Brazil: assessment by two cohort studies. Lancet. 2007;370(9599):1622–1628. 10.1016/s0140-6736(07)61638-9. 17993362 PMC2212752

[29] Fuller JA, Villamor E, Cevallos W, Trostle J, Eisenberg JN. I get height with a little help from my friends: herd protection from sanitation on child growth in rural Ecuador. Int J Epidemiol. 2016;45(2):460–469. 10.1093/ije/dyv368. 26936912 PMC5841884

[30] Van de Poel E, O’Donnell O, Van Doorslaer E. What explains the rural-urban gap in infant mortality: household or community characteristics? Demography. 2009;46(4):827–850. 10.1353/dem.0.0074. 20084831 PMC2831359

[31] Adekanmbi VT, Kayode GA, Uthman OA. Individual and contextual factors associated with childhood stunting in Nigeria: a multilevel analysis. Matern Child Nutr. 2013;9(2):244–259. 10.1111/j.1740-8709.2011.00361.x. 22004134 PMC6860873

[32] Root GP. Sanitation, community environments, and childhood diarrhoea in rural Zimbabwe. J Health Popul Nutr. 2001;19(2):73–82. 11503350

[33] Santos CA, Strina A, Amorim LD, et al. Individual and contextual determinants of the duration of diarrhoeal episodes in preschool children: a longitudinal study in an urban setting. Epidemiol Infect. 2012;140(4):689–696. 10.1017/s0950268811000690. 21676354

[34] Buttenheim AM. The sanitation environment in urban slums: implications for child health. Popul Environ. 2008;30(1-2):26–47. 10.1007/s11111-008-0074-9. 25825551 PMC4377083

[35] Adedini SA, Odimegwu C, Imasiku EN, Ononokpono DN, Ibisomi L. Regional variation in infant and child mortality in Nigeria: a multilevel analysis. J Biosoc Sci. 2015;47(2):165–187. 10.1017/s0021932013000734. 24411023 PMC4501304

[36] Kayode GA, Amoakoh-Coleman M, Agyepong IA, Ansah E, Grobbee DE, Klipstein-Grobusch K. Contextual risk factors for low birth weight: a multilevel analysis. PLoS One. 2014;9(10):e109333. 10.1371/journal.pone.0109333. 25360709 PMC4215836

[37] Sastry N. Community characteristics, individual and household attributes, and child survival in Brazil. Demography. 1996;33(2):211–229. 10.2307/2061873 8827166

[38] Barreto ML, Genser B, Strina A, et al. Impact of a citywide sanitation program in Northeast Brazil on intestinal parasites infection in young children. Environ Health Perspect. 2010;118(11):1637–1642. 10.1289/ehp.1002058. 20705544 PMC2974706

[39] Garn JV, Boisson S, Willis R, et al. Sanitation and water supply coverage thresholds associated with active trachoma: modeling cross-sectional data from 13 countries. PLoS Negl Trop Dis. 2018;12(1):e0006110. 10.1371/journal.pntd.0006110. 29357365 PMC5800679

[40] Andrés LA, Briceño B, Chase C, Echenique JA. Sanitation and externalities: evidence from early childhood health in rural India. J Water Sanitation Hyg Dev. 2017;7(2): 272–289. 10.2166/washdev.2017.143

[41] Duflo E, Greenstone M, Guiteras R, Clasen T. Toilets Can Work: Short and Medium Run Health Impacts of Addressing Complementarities and Externalities in Water and Sanitation. National Bureau of Economic Research; 2015. Accessed May 10, 2024. https://www.nber.org/system/files/working_papers/w21521/w21521.pdf

[42] Coffey D, Geruso M, Spears D. Sanitation, disease externalities and anaemia: evidence from Nepal. Econ J (London). 2018;128(611):1395–1432. 10.1111/ecoj.12491. 29937551 PMC6001781

[43] Augsburg B, Rodrigues-Lesmes PA. Sanitation and child health in India. World Dev. 2018;107:22–39. 10.1016/j.worlddev.2018.02.005

[44] Cameron L, Santos P, Thomas M, Albert J. Sanitation, financial incentives and health spillovers: a cluster randomised trial. J Health Econ. 2021;77:102456. 10.1016/j.jhealeco.2021.102456. 33857858

[45] Contreras JD, Islam M, Mertens A, et al. Influence of community-level sanitation coverage and population density on environmental fecal contamination and child health in a longitudinal cohort in rural Bangladesh. Int J Hyg Environ Health. 2022;245:114031. 10.1016/j.ijheh.2022.114031. 36058111 PMC9489923

[46] Jung S, Doh YA, Bizuneh DB, et al. The effects of improved sanitation on diarrheal prevalence, incidence, and duration in children under five in the SNNPR State, Ethiopia: study protocol for a randomized controlled trial. Trials. 2016;17(1):204. 10.1186/s13063-016-1319-z. 27089872 PMC4835836

[47] Cha S, Jung S, Bizuneh DB, et al. Effect of a community-led total sanitation intervention on the incidence and prevalence of diarrhea in children in rural Ethiopia: a cluster-randomized controlled trial. Am J Trop Med Hyg. 2021;105(2):532–543. 10.4269/ajtmh.20-0014. 34125700 PMC8437198

[48] Cha S, Jung S, Bizuneh DB, et al. Benefits and costs of a community-led total sanitation intervention in rural Ethiopia-a trial-based ex post economic evaluation. Int J Environ Res Public Health. 2020;17(14):5068. 10.3390/ijerph17145068. 32674392 PMC7399893

[49] Hayes RJ, Bennett S. Simple sample size calculation for cluster-randomized trials. Int J Epidemiol. 1999;28(2):319–326. 10.1093/ije/28.2.319. 10342698

[50] Halloran ME, Struchiner CJ, Longini IM Jr. Study designs for evaluating different efficacy and effectiveness aspects of vaccines. Am J Epidemiol. 1997;146(10):789–803. 10.1093/oxfordjournals.aje.a009196. 9384199

[51] Snijders TAB, Bosker RJ. *Multilevel Analysis.* SAGE; 2012.

[52] Larsen K, Merlo J. Appropriate assessment of neighborhood effects on individual health: integrating random and fixed effects in multilevel logistic regression. Am J Epidemiol. 2005;161(1):81–88. 10.1093/aje/kwi017. 15615918

[53] Merlo J, Yang M, Chaix B, Lynch J, Råstam L. A brief conceptual tutorial on multilevel analysis in social epidemiology: investigating contextual phenomena in different groups of people. J Epidemiol Community Health. 2005;59(9):729–736. 10.1136/jech.2004.023929. 16100308 PMC1733145

[54] Cha S, Lee JE, Seo DS, et al. Associations between household latrines and the prevalence of diarrhea in Idiofa, Democratic Republic of the Congo: a cross-sectional study. Am J Trop Med Hyg. 2017;97(2):460–468. 10.4269/ajtmh.16-0361. 28722602 PMC5544065

[55] Sinha A, Nagel CL, Thomas E, Schmidt WP, Torondel B, Boisson S, Clasen TF. Assessing latrine use in rural India: a cross-sectional study comparing reported use and passive latrine use monitors. Am J Trop Med Hyg. 2016;95(3):720–727. 10.4269/ajtmh.16-0102. 27458042 PMC5014284

[56] Schmidt WP, Chauhan K, Bhavsar P, et al. Cluster-randomised trial to test the effect of a behaviour change intervention on toilet use in rural India: results and methodological considerations. BMC Public Health. 2020;20(1):1389. 10.1186/s12889-020-09501-y. 32917160 PMC7488773

[57] Sima LC, Ng R, Elimelech M. Modeling risk categories to predict the longitudinal prevalence of childhood diarrhea in Indonesia. Am J Trop Med Hyg. 2013;89(5):884–891. 10.4269/ajtmh.12-0540. 24019442 PMC3820331

[58] Genser B, Strina A, Teles CA, Prado MS, Barreto ML. Risk factors for childhood diarrhea incidence: dynamic analysis of a longitudinal study. Epidemiology. 2006 Nov;17(6):658–667. 10.1097/01.ede.0000239728.75215.86. 17003687

[59] Clasen T, Boisson S, Routray P, et al. Effectiveness of a rural sanitation programme on diarrhoea, soil-transmitted helminth infection, and child malnutrition in Odisha, India: a cluster-randomised trial. Lancet Glob Health. 2014;2(11):e645–e653. 10.1016/s2214-109x(14)70307-9. 25442689

[60] Patil, SR, Arnold BF, Salvatore AL, et al. The effect of India’s total sanitation campaign on defecation behaviors and child health in rural Madhya Pradesh: a cluster randomized controlled trial. PLoS Med. 2014;11(8):e1001709. 10.1371/journal.pmed.1001709. 25157929 PMC4144850

[61] Luby SP, Rahman M, Arnold BF, et al. Effects of water quality, sanitation, handwashing, and nutritional interventions on diarrhoea and child growth in rural Bangladesh: a cluster randomised controlled trial. Lancet Glob Health. 2018;6(3):e302–e315. 10.1016/s2214-109x(17)30490-4. 29396217 PMC5809718

[62] Null C, Stewart CP, Pickering AJ, et al. Effects of water quality, sanitation, handwashing, and nutritional interventions on diarrhoea and child growth in rural Kenya: a cluster-randomised controlled trial. Lancet Glob Health. 2018;6(3):e316–e329. 10.1016/s2214-109x(18)30005-6. 29396219 PMC5809717

[63] Tyndale-Biscoe P, Bond M, Kidd R. ODF Sustainability Study. Plan International; 2013. Accessed May 10, 2024. https://sanitationlearninghub.org/resource/odf-sustainability-study/

[64] Humphrey JH, Mbuya MNN, Ntozini R, et al. Independent and combined effects of improved water, sanitation, and hygiene, and improved complementary feeding, on child stunting and anaemia in rural Zimbabwe: a cluster-randomized trial. Lancet Global Health. 2019;7(1):e132–e147. 10.1371/journal.pntd.0007963. 30554749 PMC6293965

