# Supplementary material for: Performance of Pit Latrines and Their Herd Protection Against Diarrhea: A Longitudinal Cohort Study in Rural Ethiopia
Source: Glob Health Sci Pract. 2024 Jun 27;12(3):e2200541. doi: 10.9745/GHSP-D-22-00541 (PMC11216697; doi:10.9745/GHSP-D-22-00541)
Supplement: GHSP-D-22-00541_supplement.pdf [file GHSP-D-22-00541_supplement.pdf]

Supplement to: Cha S, Jung S, Abera T. Performance of pit latrines and their herd protection against diarrhea: a longitudinal cohort study in rural Ethiopia. *Glob Health Sci Pract*. 2024;12(3):e2200541. https://doi.org/10.9745/GHSP-D-22-00541

Supplement 1. Details of the community-led total sanitation intervention in the Gurage zone, Ethiopia

Intervention

In alignment with the National Hygiene and Sanitation Strategic Action Plan (2011-2015), the Ethiopian Government's policy on sanitation, the principles of Community-Led Total Sanitation (CLTS) was applied. The Gurage zonal office, the SNNPR State of Ethiopia, and the Re-shaping Development Institute (ReDI) implemented the project. The project was funded by the Korea International Cooperation Agency (KOICA).

CLTS implementation

In accordance with the Ethiopian government's guidelines on this issue, the primary approach for implementation of latrine improvement and hygiene promotion adopted for this program was Community-Led Total Sanitation. Of 48 *gotts* (villages) selected in the project area, 24 *gotts* in the project area were selected (intervention arm) and received the CLTS intervention and underwent intensive follow-up throughout the first phase of the implementation period for latrine improvement. As the first step of CLTS, a team of trained CLTS facilitators conducted the triggering process in each of these 24 *gotts*. During this triggering process, facilitators used participatory tools, such as transect walk, sanitation mapping, and calculating feces deposition to help community members realize the health effects of open defecation practices in their *gotts*.

In accordance with the core principle of CLTS, no material or financial subsidies were provided for the construction of individual household latrines. Household members took responsibility for the whole process of latrine construction including (1) pit-hole digging; (2) constructing a slab and pit-hole cover; (3) constructing the walls, door, and roof; and (4) installing hand-washing facilities.

Detailed procedures

The CLTS intervention was carried out in accordance with the Ethiopian government policy from January 2016 through January 2017 (See below for details on the intervention: selection criteria, demographic profiles and core tasks of CLTS promoters; benefits, training and supervision of CLTS promoters; selection and training of CLTS facilitators; and the dates of CLTS triggering). No financial or material subsidies were provided for constructing household latrines.

A co-founder of CLTSH trained CLTS facilitators. A team of trained CLTS facilitators comprising officials from the district health office, health professionals from health centers, and health extension workers visited the villages for pre-triggering to introduce themselves to, and build rapport with, village members, and to arrange the triggering schedule.

The facilitators carried out the triggering process in the 24 intervention villages, which took one day per village between February 11 and March 18, 2016.

The core components of the CLTS triggering process were applied with the aims of having community people realize the outcomes of open defecation practices to ignite shame or disgust.

Village members walked through the village from one side to the other and visited open defecation sites and different types of latrines along the way, experiencing the disgusting sights and smells (transect walk). Village members drew a map illustrating the sanitation situation in the village, locating defecation areas and their dwellings. They were asked to discuss where the dirtiest area was in their village (defecation areas mapping). They calculated the amount of feces they produced per day, per week, per month, and per year and how much they spent for treating diarrhea, dysentery, cholera, and other diseases due to open defecation (calculations of shit and medical expenses). They were offered a glass of water in which a hair that had touched some feces was dipped, and were informed that they could ingest each other's feces via the contaminated legs of a fly (the glass of water exercise).

At the outset of the intervention, one or two people from each intervention village were selected as CLTS promoters to conduct post-triggering activities. If the number of households in a village was 70 or more, two promoters were selected. Their main task was to encourage community members to build an improved latrine in their own way, using locally available materials, through community meetings and household visits. They were recommended to visit households every week to encourage latrine uptake. In order for a latrine to protect against the transmission of fecal matter, the following components were recommended: digging a pit-hole of 2 meters depth or more; installing a slab and a pit-hole cover; constructing a wall, door, and roof; and installing a hand-washing facility with soap. We defined an improved latrine in this study as having all of these components. This is a more stringent definition of an improved latrine than that of the Joint Monitoring Program (JMP) of WHO/UNICEF.

In principle, CLTS does not prescribe toilet types. In many CLTS interventions, particularly where open defecation practices are very common, the usual approach is to convince people to build any toilet first and then continue to improve it, moving up along the sanitation ladder. However, in this trial, community members in the study areas were encouraged to build improved latrines since the coverage of simple pit latrines was already high and open defecation was not especially common like many other rural settings in sub-Saharan African countries. Materials for latrine components were not pre-specified because locally available and affordable materials could be diverse.

The CLTS facilitators and the project coordinators trained CLTS promoters for 4 days in April on how to build latrines, what latrine components are recommended, and the appropriate messages to deliver. After the training, the promoters promoted latrine improvement and followed up with the latrine construction progress. The Gurage zone office, the SNNPR state of Ethiopia, and the Re-shaping Development Institute (ReDI: a development NGO based in Korea) implemented the project.

Community-led total sanitation promoters *(CLTS promoters)*

Selection criteria for CLTS promoters

- Eligibility: CLTS promoters should be residents of the community.

- CLTS promoters who met the following criteria were recommended by community leaders or health extension workers (HEWs):

  Criterion 1: having attained a basic education level

  Criterion 2: being good at communication

  Criterion 3: having previous experience as a volunteer

- Nominated members took a written test and were interviewed by district health officials and project coordinators.

Demographic profiles of CLTS promoters

- Male: 79% (27 of 34 promoters)
- Mean age: 37 years
- Mean years of education: 8 years
- Occupation: farmer (100%)

Core tasks of CLTS promoters

- Meeting arrangements and other preparations during pre-triggering
- Active participation as 'an environment setter' during the CLTS triggering process
- Post-triggering arrangement and follow-up
- Participation in the training for capacity-building
- Follow-up on the construction of improved individual household latrines in respective *gotts* (recommended to make a weekly visit to every household: giving technical advice for construction, visiting households for sanitation promotion, and sensitizing conversations in the community)
- Participation in monthly review meetings
- Monitoring of latrine improvement progress (see Appendix for the sanitation survey form)

Benefits for CLTS promoters

- A per diem for participation was provided at every training and regular review meeting.
- They were given other training opportunities by the district health office.
- Clothes and shoes for field operations were provided.

Supplement to: Cha S, Jung S, Abera T. Performance of pit latrines and their herd protection against diarrhea: a longitudinal cohort study in rural Ethiopia. *Glob Health Sci Pract*. 2024;12(3):e2200541. https://doi.org/10.9745/GHSP-D-22-00541

Training for CLTS promoters

- All CLTS promoters were provided with 4 days of training on the topics of improved latrine construction, basics of sanitation and hygiene, and communication skills.

Supervision for CLTS promoters

- Facilitative supervision was monthly provided by the monitoring team, made up of the district health officials, health professionals from health centers, and the HEWs from health posts
- The HEW at each health post of a *gott* supervised CLTS promoters (the HEW to CLTS promoter ratio was 1:1 or 1:2).
- The monitoring team occasionally did spot checks on CLTS promoters' practices such as home visits during post-triggering.

CLTS facilitators

- HEWs from health posts, health professionals from health centers, and district health officials were selected as health facilitators (25 HEWs; 7 health professionals from health centers; and 3 district health officials).

Training for CLTS facilitators

- All CLTS facilitators were provided with 5 days of training, including 2 days of field-based practice (trainer: Dr. Dawit Belew Bizuneh: a co-founder of CLTSH in Ethiopia).
- Pre- and post-test results of CLTS facilitators' training: (pre-test score of pass [i.e., 70 or more): 2 of 34; post-test: 22 of 34)
- CLTS facilitators with a poor score on the post-training test were given additional training.

CLTS triggering

- CLTS triggering was conducted from February 11 to March 2, 2016 in the Enemore Ena district and from March 8 to 18, 2016 in the Cheha district.

Primary and secondary outcomes (fidelity)

The primary endpoint of the study is diarrheal prevalence in children under 5 years of age. We used 7-day prevalence of reported diarrhea in the household, which were based on parental reports. In addition to diarrheal prevalence, we measured diarrheal incidence and diarrheal duration. For documentation of diarrheal incidence and duration, we had the mother or caregiver record the diarrheal episodes of her youngest child on a "Sanitation Calendar" (Amharic language: Yenezehenna Gize Saleda) on a daily basis.

Supplement to: Cha S, Jung S, Abera T. Performance of pit latrines and their herd protection against diarrhea: a longitudinal cohort study in rural Ethiopia. *Glob Health Sci Pract*. 2024;12(3):e2200541.
https://doi.org/10.9745/GHSP-D-22-00541

There were 202 cases (481 days of diarrhea) in the intervention group and 298 cases (773 days of diarrhea) in the control group during the 140 days of follow-up. The 7-day period prevalence of child diarrhea based on caregiver's recall decreased from 22.2% at baseline to 11.8% at the 3-month follow-up and 7.7% at the 10-month follow-up in the intervention group. The prevalence rose from 17.1% at baseline to 17.2%, and declined to 9.9% at the same time points in the control group.

The secondary outcomes were defined as latrine coverage and latrine use. An intermediary outcome of fecal-oral contamination was also assessed by counting the number of feces inside and outside of the household compound, and assessing the number of flies (using a glue trap placed adjacent to the pit-hole for 30 minutes). Latrine construction status was directly observed by enumerators. Each component of the latrine structures (pit, slab, pit-hole cover, wall, roof, door, and hand-washing facility) were photographed by enumerators in every round of the survey. To assess whether the latrine was being used or not, enumerators checked for the presence of a worn path to the latrine, spider webs at the entrance, fresh feces inside the pit, and odor. In addition, direct observations were made of the presence of human feces inside and outside of the household compound. The mean proportion of households with an improved latrine increased from 0.0% at baseline to 35.0% at 10 months after the CLTS triggering in the intervention villages, while it increased from 0.5% to 2.8% in the control villages. At the 10-month follow-up, four of the 24 intervention villages had improved latrine coverage of 70% or greater. Meanwhile, in the control villages, no community had a coverage of 30% or greater. Ownership of a partially improved household latrine (defined as having a pit, pit-hole cover, and slab) in this study rose from 11.9% at baseline to 69.0% at 10 months after the CLTS triggering in the intervention group, compared with the corresponding rates of 11.6% at baseline and 15.0% at follow-up in the control group. The coverage of any type of latrine was already high at baseline and continued to increase in both arms. Based on the results of direct observations, the coverage of any type of latrine at baseline in the intervention villages was 70.3% and increased to 99.5% 10 months after the CLTS triggering, and the corresponding values in the control group were 75.8% at baseline and 90.8% at 10 months of follow-up. All caregivers who had any type of latrine in both the intervention and control groups reported that they were using the latrine at the 10-month follow-up (99.5% vs 90.8% in the intervention and control group, respectively.).

Diarrhea calendar

- The diarrhea calendar was distributed from May 30 through June 10, 2016.

Supplement to: Cha S, Jung S, Abera T. Performance of pit latrines and their herd protection against diarrhea: a longitudinal cohort study in rural Ethiopia. *Glob Health Sci Pract*. 2024;12(3):e2200541. https://doi.org/10.9745/GHSP-D-22-00541

Supplement Table S1. Performance of latrines on child diarrheal prevalence by type

		Having a latrine but not up to JMP improved	Having a JMP improved latrine (a)		Presence of a Study-improved latrine (b)	
			**unadjusted**	**adjusted**	**unadjusted**	**adjusted**
**All**	**OR**		**0.87**	**0.99**	**0.46**	**0.46**
	**95% CI**		**0.51-1.49**	**0.56-1.79**	**0.26-0.80**	**0.27-0.81**
	**p-value**		**0.62**	**0.99**	**0.006**	**0.006**
June (3 months)	n/N	25/182		75/481		8/102
	%	13.74%		15.59%		7.84%
	OR		1.75	2.05	0.27	0.26
	95% CI		0.56-5.41	0.58-7.24	0.05-1.32	0.04-1.51
	p-value		0.33	0.27	0.11	0.13
December (9 months)	n/N	1/8		75/647		8/127
	%	12.50%		11.59%		6.30%
	OR		0.91	0.86	0.50	-
	95% CI		0.00-2399.99	0.01-143.55	0.03-8.83	-
	p-value		0.98	0.95	0.64	-
January (10 months)	n/N	0/5		62/674		7/138
	%	0.0%		9.20%		5.07%
	OR			-	-	-
	95% CI			-	-	-
	p-value			-	-	-

a Reference: Those with a latrine but not up to a JMP improved latrine (adjusted for individual variables: child's age and sex, presence of improved water source, handwashing behavior at four critical times)
b Reference: Those with a JMP improved latrine (adjusted for individual variables: child's age and sex, presence of improved water source, handwashing behavior at four critical times)

Supplement to: Cha S, Jung S, Abera T. Performance of pit latrines and their herd protection against diarrhea: a longitudinal cohort study in rural Ethiopia. *Glob Health Sci Pract*. 2024;12(3):e2200541. https://doi.org/10.9745/GHSP-D-22-00541

Supplement Table S2. Comparison of performance between **unimproved** latrines **in high- and low-coverage areas, and improved latrines in high-coverage areas** and **unimproved latrines in low-coverage areas (based on the coverage 50%of an improved latrine coverage)**

		Low coverage		High coverage			Comparison of **unimproved** latrines in **high- and low-coverage areas (herd protection)**			Comparison of **improved latrine in high-coverage areas** and **unimproved latrine in low-coverage areas**		
		Absence of a latrine	Presence of a latrine, but not an improved one	Absence of a latrine	Presence of a latrine, but not an improved one	Improved latrine	OR	95% CI	p-value	OR	95% CI	p-value
**All**							**0.30**	**0.06-1.61**	**0.16**	**0.32**	**0.08-1.30**	**0.11**
June	n/N	10/57	100/646	0	0/17	3/37	0.50	0.24-1.06	0.07	0.59	0.20-1.77	0.35
	%	17.5%	15.5%		0.0%	8.1%						
December	n/N	9/42	75/631	0	1/24	4/63	0.43	0.05-3.55	0.43	0. 37	0.08-1.67	0.20
	%	21.4%	11.9%		4.2%	6.4%						
January	n/N	7/40	59/626		3/53	3/105	0.78	0.20-3.04	0.72	0.19	0.01-2.43	0.20
	%	17.5%	9.4%	0	5.8%	2.9%						

Supplement to: Cha S, Jung S, Abera T. Performance of pit latrines and their herd protection against diarrhea: a longitudinal cohort study in rural Ethiopia. *Glob Health Sci Pract*. 2024;12(3):e2200541. https://doi.org/10.9745/GHSP-D-22-00541

Supplement Table S3. Detailed status of JMP improved latrines (% with the following component)

	Drop-hole cover	wall	roof	door	2 or more
3 months	48.5%	77.0%	66.6%	42.2%	61.0%
9 months	45.5%	83.1%	73.4%	56.6%	52.1%
10 months	44.8%	83.1%	74.8%	56.2%	51.2%
